# Improving Post-PCI Cardiac Rehabilitation Referrals: A Collaborative Approach

**DOI:** 10.1016/j.jaccas.2025.105770

**Published:** 2025-10-07

**Authors:** Sreedeepa Raveendran, Duane Bruce, Esperanza Avena, Aditya Mangla, Robert Mendelson, Zoran Lasic

**Affiliations:** a Division of Cardiology, Jamaica Hospital Medical Center, Queens, New York, USA; b Division of Cardiology, Lenox Hill Hospital, Northwell Health, New York, New York, USA

**Keywords:** cardiac rehabilitation, lifestyle, percutaneous coronary intervention

## Abstract

**Background:**

Clinical practice guidelines recommend referring all eligible patients who have undergone percutaneous coronary intervention (PCI) to cardiac rehabilitation (CR).

**Project Rationale:**

The COVID-19 pandemic severely decreased patient participation in CR programs after PCI. Many CR facilities either closed or paused their services, and this decline in participation continued even after the pandemic ended.

**Project Summary:**

The hospital implemented a new process to improve referrals for CR, which included providing daily education to patients who had undergone PCI, submitting a CR referral request before the patient was discharged, having a representative from the CR facility contact the patient directly, and giving patients a choice of multiple locations for their CR. Within the first quarter, the referral rate doubled, and for the next 7 quarters, the rate consistently stayed above 97%.

**Take-Home Message:**

Collaboration with a dedicated CR facility leads to improved and sustained referral rates among patients who have undergone PCI.

## Graphical Abstract

**Figure:**
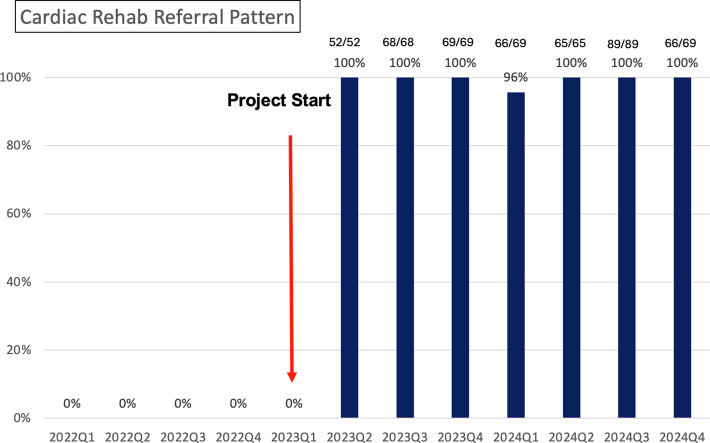

Clinical practice guidelines strongly recommend that all eligible patients who have undergone percutaneous coronary intervention (PCI) be referred to a cardiac rehabilitation (CR) program.^1^ This referral is crucial and should be initiated either before the patient leaves the hospital or during their very first outpatient visit. The primary goals of this early and consistent referral are to enhance healthy lifestyle habits, improve key health metrics, reduce the likelihood of hospital readmissions, and improve survival.^2^

**Take-Home Message**

• Collaboration with a dedicated cardiac rehabilitation facility leads to improved and sustained referral rates among patients who have undergone percutaneous coronary intervention.

CR is a medically supervised program that includes exercise training, education on heart-healthy living, and counseling to reduce stress and other risk factors. By connecting patients with these programs early, health care providers can ensure they receive the comprehensive support needed to make lasting changes that improve their long-term heart health.

## Project Rationale

The COVID-19 pandemic caused a major drop in participation in CR programs for patients who had undergone PCI. This was largely due to many CR facilities either closing or temporarily stopping their services.

Unfortunately, this trend did not end with the pandemic. The reduced patient enrollment has continued to be a significant challenge, making it difficult to provide the comprehensive, long-term care that patients who have undergone PCI need for a full recovery and improved quality of life.

## Project Description

Jamaica Hospital Medical Center, a community hospital in Queens, New York, holds the designation of Primary Heart Attack Center from the Joint Commission. We are also active participants in the NCDR's CathPCI and CPMI registries, demonstrating our commitment to national quality standards.

Recognizing the need to improve CR referrals, we undertook extensive research to identify suitable CR facilities in our area. Our primary goal was to find partners with multiple convenient locations and broad insurance acceptance, ensuring our patients could easily access the care they need.

After a thorough selection process, leadership from both our hospital and the chosen CR facilities collaborated to establish a new, streamlined referral process.

## Project Deliverables

The new protocol was designed to be as efficient as possible and is outlined below.

### New CR referral process

• Daily patient education: All patients who had undergone PCI received daily education about the benefits of CR from their health care providers. This ensured that the patients are fully informed and understand the importance of CR for their long-term recovery.
• Timely referral: A formal CR referral request was completed and submitted for each patient before they were discharged from the hospital. This proactive step ensured there was no delay in connecting patients with the resources they need.
• Direct patient contact: A representative from the CR facility proactively contacted the patient directly to arrange their first appointment and answer any questions. This helped to overcome barriers to enrollment and provided a smooth transition from hospital care to outpatient rehabilitation.
• Patient choice: To empower patients and increase the likelihood of participation, they were given a list of multiple convenient CR facility locations to choose from, allowing them to select the one that best fits their needs.

## Project Outcome

**Figure 1:**
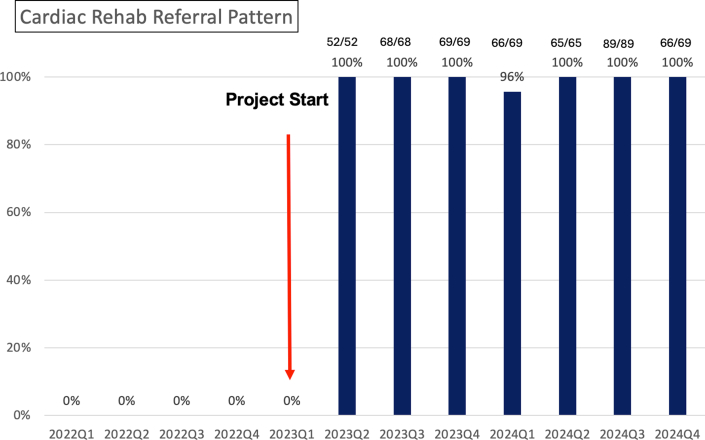
Cardiac Rehabilitation Referral Pattern From the First Quarter of 2022 to the Fourth Quarter of 2024 Ratios above the columns represent the number of patients referred to cardiac rehabilitation divided by the total number of patients.

After the implementation of the new referral process in the first quarter of 2023, the number of patients referred to CR increased dramatically. Within just 1 quarter, the number of CR referrals doubled, reaching a 100% increase (Figure 1).

Since the beginning of this program, we have closely monitored the referral rates. For the 7 subsequent quarters, the rates consistently remained above 96%, demonstrating the remarkable and sustained success of this new process. This significant improvement ensured that more patients received the comprehensive care they need for a successful recovery.

## Discussion

Our partnership with a dedicated CR facility was a key factor in developing an effective and sustainable referral process for our patients who had undergone PCI. This collaboration has not only streamlined the patient journey but has also proven to be a long-term solution. By working together, we were able to create a process that ensures patients receive consistent and timely access to the rehabilitation they need for their recovery, demonstrating a lasting impact on patient care.

## Conclusions

In the post-COVID-19 era, ensuring that patients who have undergone PCI are referred to CR remains a significant challenge. However, a strategic partnership with a dedicated CR facility proved to be a key solution. This collaboration was instrumental in developing an effective and sustainable referral process that continues to benefit patients long after the pandemic. By working together, we were able to create a streamlined system that addresses the existing barriers and ensures patients receive the continued care they need.

## Funding Support and Author Disclosures

The authors have reported that they have no relationships relevant to the contents of this paper to disclose.
